# Population Management: A Tool to Improve Timely Care in Pediatric and Young Adult Patients with Inflammatory Bowel Disease

**DOI:** 10.1155/2019/4702969

**Published:** 2019-07-18

**Authors:** Erealda Prendaj, Sharon Thomas, Gitit Tomer

**Affiliations:** Division of Pediatric Gastroenterology, Hepatology, and Nutrition, Children's Hospital at Montefiore, The University Hospital for Albert Einstein School of Medicine, Bronx, NY, USA

## Abstract

**Background:**

Maintenance of health leads to better outcomes in patients with chronic illness. ImproveCareNow, an international inflammatory bowel disease (IBD) quality improvement (QI) network, recommends maintenance-of-health visits twice a year. We identified a gap in care, with only 64% of IBD patients having documented visits within 200 days. Therefore, we sought to improve our follow-up rate to a goal of 80%.

**Methods:**

Using population management (PM) reports, we identified patient-, data-, and treatment-related reasons for no documented visit within 200 days. We used the Pareto chart, key drivers, and process flow mapping and implemented changes using Plan-Do-Study-Act (PDSA) cycles to improve follow-up visit rates. Outcomes were presented using a control run chart with pre- and post- intervention data.

**Results:**

The most common reasons for no visits were patient nonadherence with appointments (50%) and relocation/transition to an adult provider (25%). The median percentage of documented visits within 200 days increased from 64% to 83% (*p* < 0.0001), and this increase has been sustained for one year.

**Conclusions:**

Using the PM tool and focused QI interventions improved data quality and the percentage of patients with a documented visit within 200 days. The process is simple and can be applied to patients with other chronic illnesses.

## 1. Introduction

Inflammatory bowel diseases (IBD), including Crohn's disease (CD) and ulcerative colitis (UC), are immune-mediated, chronic inflammatory diseases of the gastrointestinal tract that have a waxing and waning clinical course and result in significant morbidity in pediatric patients. There are about 1.3 million individuals suffering from IBD in the United States [1], with about 15-25% of the IBD patients diagnosed before 18 years of age [2, 3]. The goal of therapy is to induce remission, maintain remission, and improve quality of life [4].

It has been well documented in the literature that preventive care and maintenance of health lead to improved patient outcomes. Patients who receive care regularly from their primary care provider are less likely to utilize emergency departments (ED) or be admitted to the hospital [5]. Similarly, IBD patients are less likely to go to the ED if they have seen their gastroenterologist within the year [6], and patients with IBD often consider their gastroenterologist to be their primary care provider. The frequency of office visits depends on disease activity and therapeutic regimen, but regular office visits at least twice a year are recommended even when patients are well, in order to provide the maintenance of health and monitor for drug toxicity [4, 7, 8].

ImproveCareNow (ICN) is a collaborative health community and an international quality improvement network, where clinicians, researchers, patients, and parents at more than 90 centers work together to improve the health and care of pediatric IBD patients [9–11]. The ICN network developed Model IBD Care Guidelines [7], defined key measures to assess performance, built a robust database, and created rigorous reports to identify gaps in care to improve the quality of care [10]. IBD patients who are enrolled in the ICN network have their demographic data, disease activity, nutrition, growth, medication, and laboratory tests recorded at each visit. This aggregation of patient data is compiled into population management (PM) reports, enabling ICN centers to identify individual patients within the groups and use that information to improve health outcomes for these patients [9]. The ICN Model of Care Guidelines recommend health supervision visits every 6 months. Visits are tracked as a key measure; the percent of patients with a documented visit within 200 days for each center and specific patient data can be extracted via population management reports.

The division of Pediatric Gastroenterology at the Children's Hospital at Montefiore (CHAM) joined ICN in 2012 and started enrolling patients in January of 2013. Analysis of CHAM key quality measures revealed that from April 2015 to September 2015, there was a decrease in the percentage of patients who had a documented visit within 200 days to 64%, which was below the ICN network goal of 80%. In October 2015, a quality improvement (QI) team was assembled, with the SMART (specific, measurable, achievable, relevant, and time bound) aim to increase the percentage of IBD patients with a documented visit within 200 days using a population management approach. The global aim was that improving timely visits will improve the maintenance of health and care.

## 2. Materials and Methods

### 2.1. Forming the QI Team

This QI project was conducted at CHAM, a quaternary children's hospital with 106 inpatient beds, 9,000 admissions a year, and 60,000 annual ED visits. CHAM is the pediatric hospital for the Albert Einstein College of Medicine located in the Bronx, New York. The pediatric GI division of Pediatric Gastroenterology and Nutrition at CHAM consists of twelve pediatric gastroenterologists, four nurse practitioners, four registered nurses, and six pediatric gastroenterology fellows. The ICN team is trained in the Model for Improvement [12], quality improvement tools, and is encouraged to audit data. A multidisciplinary QI team was formed, composed of two physicians, an IBD nurse practitioner, a research coordinator, and an administrative assistant.

### 2.2. PM Report: Visit within 200 Days

CHAM IBD patients who are enrolled in the ICN network have their demographic data, disease activity, nutrition, growth, medication, and laboratory tests recorded via structured data forms at each visit. These data are manually entered into an ICN web-based FDA-approved clinical data registry. This aggregation of patient data generates monthly key measures and population management reports, which contain specific information for CHAM performance.

The ICN PM report allows CHAM and other centers to examine, in detail, the care provided to its entire IBD population. Data from the ICN centralized registry are used to create a Microsoft Excel-based interactive report that provides comprehensive patient information across multiple categories (e.g., number of patients in remission, patients with growth or nutritional failure, and visit within 200 days). By selecting the “visit within 200 days” tab, all of the patients without a visit within 200 days were identified, along with multiple clinical variables pertaining to these patients (e.g., current diagnosis and PGA) [9]. This information allowed ongoing, targeted intervention and monitoring of patients who had no documented visit within 200 days.

### 2.3. Objectives

**Figure 1:**
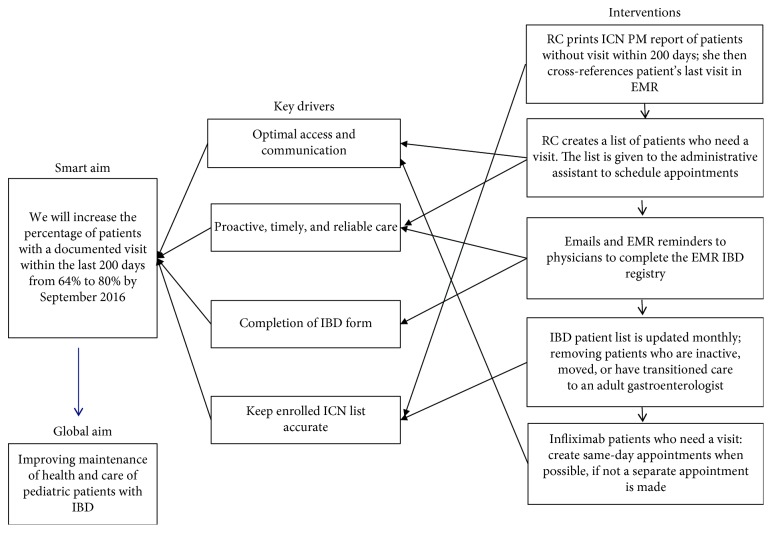
Key driver diagram. Abbreviations: ICN: ImproveCareNow; RC: research coordinator; PM: population management; EMR: electronic medical record.

**Figure 2:**
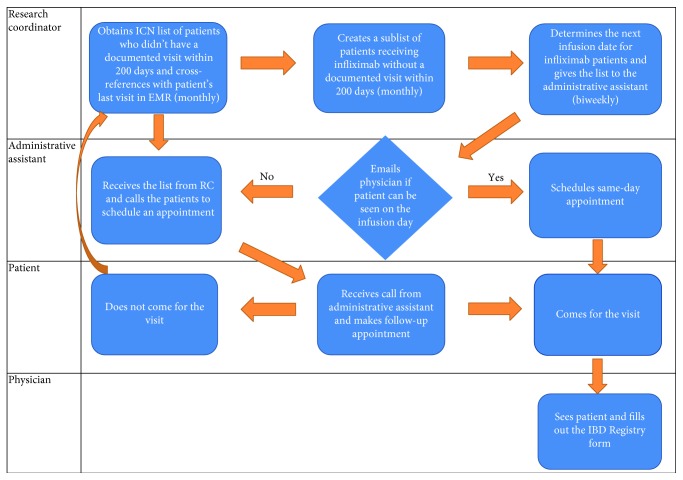
Process flow map. Abbreviations: ICN: ImproveCareNow; RC: research coordinator; EMR: electronic medical record.

As previously noted, by September 2015, only 64% of ICN-enrolled IBD patients had a documented visit within 200 days. Our SMART aim was that by September 2016, we would increase follow-up rates, defined as a visit within 200 days, from a median of 64% to 80%. To achieve this objective, we started printing, reviewing, and analyzing the ICN PM reports monthly. We identified patients without a documented visit within 200 days. The reasons for lack of documented visit within 200 days were determined. Based on the reasons for poor follow-up, key drivers and primary process intervention steps were developed (Figure 1). Interventions were implemented as Plan-Do-Study-Act (PDSA) cycles and included maintaining an accurate active IBD patient list, calling patients to schedule appointments monthly, and conducting office visits during the time of infliximab infusion for infliximab patients (which was not routinely done at our center prior to this study). The process flow map summarizes these interventions (Figure 2).

### 2.4. Interventions

#### 2.4.1. Population Management and Data Quality

PDSA cycle 1 focused on maintaining an accurate active IBD patient list. After the initiation of this quality improvement study, at the beginning of each month, the research coordinator (RC) printed an ICN PM report of patients who were not seen within 200 days. She then reviewed electronic medical records to check if patients indeed did not have visits. This PDSA cycle revealed that the three main reasons for lack of visits within 200 days are as follows: (1) visits occurred but were not captured in the ICN database due to the IBD form not being completed by physicians (for intervention, please see PDSA cycle 2); (2) visits occurred and physicians completed the IBD form, but the RC was not aware that the visit took place (due to last minute add-on visits) and she then entered the visit data into the ICN database; (3) patients had moved or transferred care to adult providers, and she then inactivated these patients from the ICN registry.

PDSA cycle 2 addressed patients who had a visit but the IBD form was not filled by the physician; therefore, the visit could not be captured in the ICN database and the patient appeared in the “no visit in 200 days” list. The RC emailed/sent electronic medical record (EMR) messages to physicians individually, and as a group, to remind them to fill out the IBD forms.

#### 2.4.2. Patient Phone Calls

PDSA cycle 3 focused on calling patients who needed to be seen and offering them appointments. At the beginning of every month, the RC or the ICN team physician supplied the administrative assistant with the updated list of IBD patients who needed to be seen, and she then reached out to these patients and scheduled them for an office visit.

#### 2.4.3. IBD Team and Patient Education

Another intervention taken was an educational intervention to increase awareness within our division of the recommended visit frequency based on ICN Model of Care Guidelines. This was achieved through lectures given by the QI team physicians about ICN Model of Care Guidelines, the project itself including baseline data, reasons for lack of documented visits within 200 days, interventions, and the progress we made. Physicians also educated their patients of the need to be seen at a minimum twice a year for the maintenance of health.

#### 2.4.4. Infliximab Infusion and Clinic Visit on the Same Day

In PDSA cycle 4, we identified that a significant number of infliximab patients did not have a visit within 200 days. Therefore, the following interventions were implemented: (1) the RC created a list of the infliximab patients who needed to be seen and determined when their next infliximab infusion was scheduled. She then gave this list to the administrative assistant who reached out to the physicians to determine if the patient could also have a clinic visit on the day of their infliximab infusion. (2) All infliximab infusion appointments were sent to physicians' outlook calendars as a reminder that the patient is having infusion that day. (3) The nurse administering the infusion would call or text the patient's physician on the day of their infusion alerting them that the patient was receiving the infusion and asking the physician if they needed to see the patient. This served as another reminder for physicians. (4) If a same-day appointment could not be offered, then an appointment was scheduled for another day.

### 2.5. Data Analysis

Data was collected from the ImproveCareNow database, from April 2015 to September 2016. This study was approved by the Albert Einstein College of Medicine Institutional Review Board, the Bronx, New York. A *t*-test and Fisher's exact test were used to analyze differences in categorical variables. A *p* value less than 0.05 was considered statistically significant. Process and outcome measures were analyzed by using an *x*-bar statistical process control chart.

## 3. Results

From January 2013 to September 2016, 137 patients with IBD have been enrolled in ICN at CHAM. Over the years, as patients have relocated or transferred care to adult providers, they have been inactivated from the ICN registry; thus, there were only 84 active IBD patients in the CHAM ICN registry at the time this manuscript was prepared. The demographics, disease characteristics, and treatment regimens of these patients are summarized in Table 1. The majority of the patients are from minority groups, reflecting the heterogeneous population of the community.

**Figure 3:**
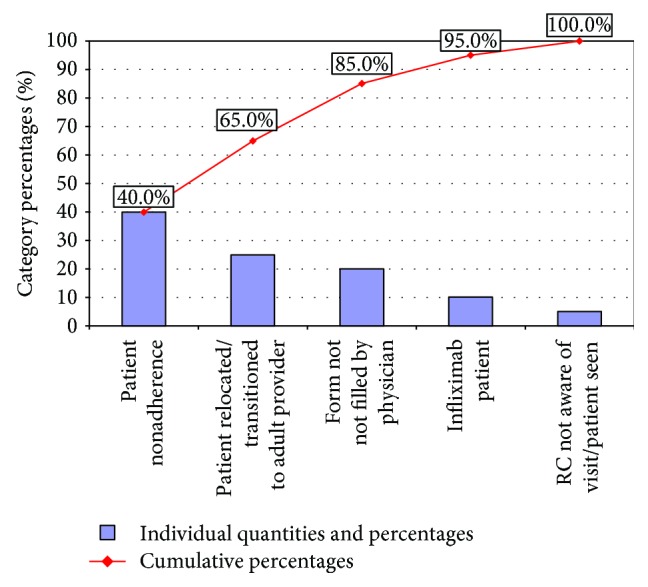
Pareto chart, reasons for no visit in 200 days. Abbreviation: RC: research coordinator.

In the six months preceding the formation of the QI team (April 2015–September 2015), the percentage of IBD patients with a documented visit within 200 days (as reflected in the ICN registry) decreased from 74% to 61% (median, 64%). We used a PM report to identify patients without a documented visit within 200 days and then reviewed patients' medical records. The most common reasons for no visit within 200 days were patient nonadherence with visits (50%) and relocation/transition to an adult provider (25%), as depicted in the Pareto chart in Figure 3.

**Figure 4:**
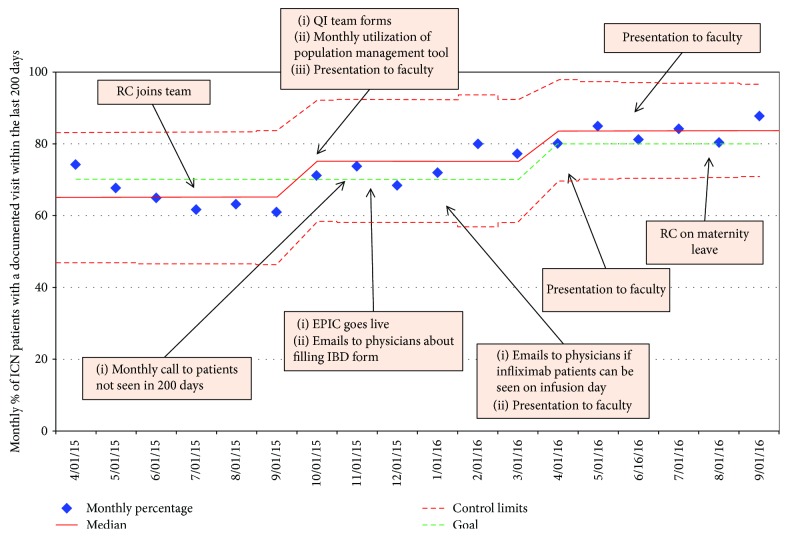
Percentage of IBD patients with a documented visit within 200 days. Run chart showing the percentage of IBD patients with a visit within the last 200 days. The data points from April to September are “pre-intervention,” and the subsequent data is “post-intervention.” The rectangles detail the interventions taken at different time points. The perforated red lines indicate upper and lower limits. Abbreviations: RC: research; QI: quality improvement.

In the 12 months postimplementation of PDSA cycle interventions, documented visits within 200 days increased to 83% (range of 68-88% per month). This improvement is statistically significant (*p* < 0.0001), and it is depicted in a control run chart (Figure 4).

Since the “documented visits” within 200 days represent visits captured in the ICN registry, we did an additional analysis of the number of office visits in the pre- and post-intervention periods. In the pre-intervention period, 72% of patients had an office visit within 200 days, as compared to 87% of patients in the post-intervention period (*p* = 0.027). The patients on infliximab also had a notable increase in follow-up rates, from 67% in the pre-intervention period to 81% in the post-intervention period; however, this increase was not statistically significant due to the small numbers of patients in this subset.

## 4. Discussion

This quality improvement project demonstrates that utilizing PM systematically and implementing simple QI measures result in a significant improvement in IBD patient follow-up rates, as well as in data quality. Timely and reliable care is essential when striving to provide better care and to maintain these complex patients in remission. Following evidence-based treatment guidelines and minimizing variation in care have led to improved outcomes in patients with chronic diseases, hence the focus of these QI efforts in following ICN Model of Care Guidelines [13–15].

Utilization of ICN PM reports allowed us to identify important patient data and treatment-related reasons for lack of documented visits within 200 days (Figure 3). Patients may have several reasons for nonadherence with visits, including forgetfulness, feeling well, financial reasons, lack of awareness of necessary visit frequency, and time constraints. Using ICN PM reports, we identified patients who missed their appointments or did not have scheduled appointments. The IBD program administrator called the patients and provided them with new appointments. Another important finding was that patients receiving infliximab were not seen routinely, and some had no visits for more than year. Patients receiving infliximab have moderate to severe disease and need to be seen at least twice a year, if not more, to monitor their weight gain, growth, and response to treatment and to ensure maintenance of health. IBD patients who are on infliximab come to the hospital a minimum of six times a year for their infliximab infusions, and additional visits can be time consuming and costly (additional copay, travel cost, parking, and time off from school/work). We have accommodated our IBD patients and created same-day office visits before or after the infusion when possible, which increased infliximab patient visits to 81%. We suspect that this improvement was not statistically significant due to the small number of patients in this group.

Using PM reports on a regular basis also created an audit of the data, ensuring that patient visits were captured in ICN and physicians filling the EMR IBD forms. We also set up lectures educating the physicians and nurses in the GI division that patients needed to be seen twice a year for the maintenance of health; these lectures facilitated buy-in from providers. Additionally, we have been educating our IBD patients and families and have made them aware that the IBD guidelines recommended for them to be seen at least twice a year for the maintenance of health, and most were receptive.

The PM tool is simple and can be implemented and sustained not only by other gastroenterology practices but also by any practice that follows patients with chronic illnesses. In fact, Woodridge et al. used a tracking system for CF patients to improve their adherence to care guidelines [16]. Our division is part of ICN; thus, we have access to PM reports, which allows us to quickly track our patients, identify those who were lost to follow-up, and call them to schedule an appointment. Our process can also be implemented by other practices by using population management through their electronic health medical records and then making interventions such as the ones we found to be very effective.

One limitation of this project is the relatively small number of patients. In practices that have higher numbers of patients, the time that the administrative assistant will have to spend making phone calls and scheduling visits will clearly increase, thus affecting workload and raising the cost of this intervention. Another limitation is that this is not an automatic process; although the ICN PM report of those who were not seen within 200 days can be generated at any time, it needs to be cross-referenced with the electronic medical record to ensure that those patients reported in the ICN PM tool indeed did not have a visit, before calling them to schedule new appointments. This process can be time consuming and will be overcome partially by the automatic transfer of the data between an EMR and the ICN database. When this automatic transfer is implemented, all visits with completed IBD forms would be captured in the registry automatically, making missed visits by the RC obsolete. However, the RC will still need to review records of patients who were not seen and try to determine the reason why. Once the systems are in place and the project is in its sustaining phase, it should not take more than 1 to 2 hours a month to continue the interventions.

## 5. Conclusion

We have demonstrated that using the PM tool and focused QI intervention resulted in a successful process in improving IBD patients' follow-up rates and data quality. The process is simple and can be applied to any patients with chronic diseases that require regular follow-up visits to improve care and maintenance of health.

## Figures and Tables

**Table 1:** CHAM IBD patient demographics.

Variable	No. (%)
Age	
0-5	1 (1%)
6-10	7 (8%)
11-14	13 (15%)
15-17	24 (29%)
18-21	39 (46%)
Gender	
Female	34 (40%)
Male	50 (60%)
Race/ethnicity	
Hispanic	30 (36%)
White	22 (26%)
Black	17 (20%)
Asian	2 (2%)
Pacific Islander	1 (1%)
Multiracial	1 (1%)
Not disclosed	11(13%)
Diagnosis	
Crohn's	55 (65%)
Ulcerative colitis	25 (30%)
Indeterminate	4 (5%)
Treatment	
Anti-TNF	31 (37%)
Immunomodulator	30 (35%)
5-ASA	18 (21%)
Other	5 (6%)

## Data Availability

The data used to support the findings of this study are available from the corresponding author upon request.
